# Truncating Variants in Titin Independently Predict Early Arrhythmias in Patients With Dilated Cardiomyopathy

**DOI:** 10.1016/j.jacc.2017.03.530

**Published:** 2017-05-16

**Authors:** Upasana Tayal, Simon Newsome, Rachel Buchan, Nicola Whiffin, Roddy Walsh, Paul J. Barton, James S. Ware, Stuart A. Cook, Sanjay K. Prasad

Dilated cardiomyopathy (DCM) has a population prevalence of ∼1 in 500 and is associated with prognostically adverse arrhythmias at initial disease presentation in up to one-third of patients (1). While increasing age, male sex, and impaired ventricular function are established arrhythmic risk factors, arrhythmias also occur in patients without known risk factors.

Recently, the advent of high throughput sequencing technologies has enabled new insights into the genetic predisposition of DCM. In particular, titin-truncating variants (TTNtv) are now known to occur in ∼15% of cases of DCM and represent the commonest genetic cause of DCM 2, 3. We evaluated whether genetic information can be used as an additional tool to identify patients at risk for arrhythmias by exploring whether there is an association between TTNtv and the occurrence of arrhythmias at the time of first diagnosis in a large cohort of patients with DCM.

In total, 572 prospectively recruited patients fulfilling diagnostic criteria for DCM by cardiovascular magnetic resonance were recruited between 2009 and 2015 (68% men, mean age 53.5 ± 14.4 years). All patients had detailed clinical assessment and sequencing for novel or rare (Exome Aggregation Consortium frequency <0.001) truncating variants in constitutively expressed TTN exons. Focusing on early arrhythmic risk, data on arrhythmia history (atrial fibrillation [AF], nonsustained ventricular tachycardia [VT], and sustained VT) on recruitment to the study were collated from hospital and primary care notes. Multivariable logistic regression was used to evaluate variables associated with arrhythmias at presentation.

In the cohort, mean left ventricular ejection fraction was 39.0 ± 12.6% (median = 40%; interquartile range: 29% to 49%). Midwall late gadolinium enhancement (LGE) myocardial fibrosis was detected in 198 patients (35%). A family history of DCM was found in 82 patients (14%) and a family history of sudden cardiac death in 76 patients (13%).

Arrhythmias prior to recruitment were documented in 196 (34%) patients. Specifically, 139 (24%) patients had confirmed AF, 69 (12%) patients had confirmed nonsustained VT and 11 (2%) patients had confirmed sustained VT. Of these, 22 patients had more than 1 type of arrhythmia: 15 had both AF and nonsustained VT; 1 had both AF and sustained VT; 5 had both sustained VT and nonsustained VT; and 1 had AF, nonsustained VT, and sustained VT.

Patients with arrhythmia were more likely to be older, be men, and have worse biventricular function (age 58.7 ± 12.2 years vs. 50.8 ± 14.7 years; 161 [82.1%] men vs. 227 [60.4%] men; left ventricular ejection fraction 36.1 ± 12.1% vs. 40.4 ± 12.7%; right ventricular ejection fraction 33.9 ± 13.7% vs. 40.7 ± 13.8%; p < 0.0001 for all). Although LGE is associated with arrhythmia later in established disease, there was no significant difference in the proportion of patients with LGE between the arrhythmia positive and negative groups at presentation (122 [32.4%] vs. 76 [38.8%]; p = 0.16).

TTNtv were observed in 13.3% (n = 26) of patients with a history of arrhythmia compared to 8% (n = 30) of patients without a history of arrhythmia (p = 0.05). Conversely, an arrhythmia was documented in 26 patients (46%) with TTNtv compared to 170 patients (33%) without TTNtv (p = 0.05). In exploratory univariable analysis, the presence of a TTNtv was predictive of baseline arrhythmia in DCM patients (unadjusted odds ratio: 1.76; 95% confidence interval: 1.01 to 3.08; p = 0.05) (Table 1).

This association was stronger in multivariable regression analyses, adjusting for variables associated with baseline arrhythmia in this cohort (age, gender, indexed left atrial volume, and ventricular function). TTNtv independently predicted early arrhythmias in DCM (adjusted odds ratio: 2.90; 95% confidence interval: 1.48 to 5.78; p = 0.002) (Table 1).

Variants in *LMNA* are found in up to 4% of DCM cases and are strongly associated with an arrhythmic phenotype. In sensitivity analyses to control for potential *LMNA* effects, TTNtv remained predictive of arrhythmia after 12 patients with rare (Exome Aggregation Consortium frequency <0.001), protein-altering *LMNA* variants were excluded from analysis (adjusted odds ratio: 2.88; 95% confidence interval: 1.44 to 5.81; p = 0.003). Putative DCM variants in other genes were not evaluated due to the small number of affected individuals and no prior associations with arrhythmia.

Our data demonstrate that TTNtv are associated with early arrhythmic risk in patients with DCM, independent of conventional arrhythmic risk factors. Although all patients were identified prospectively, baseline arrhythmia data were collected retrospectively and we have consolidated ventricular and atrial arrhythmias into 1 arrhythmia category, with a modest absolute increase in arrhythmic risk (13%). However, these findings have relevance for all DCM cases with TTNtv, representing ∼15% of all DCM. This study provides insights into the arrhythmic burden associated with TTNtv and highlights additional genetic tools for the stratification of high-risk DCM patients.

## Figures and Tables

**Table 1:** Results of Logistic Regression Analysis

Variable	Unadjusted Analysis			Adjusted Analysis		
	OR	p Value	95% Confidence Interval	OR	p Value	95% Confidence Interval
Age (per 10 yrs)	1.53	<0.0001	1.33–1.76	1.60	<0.0001	1.36–1.89
Male	3.02	<0.0001	2.00–4.65	2.33	<0.0001	1.44–3.83
LVEF (per 10%)	0.76	<0.001	0.66–0.87	—	—	—
RVEF (per 10%)	0.71	<0.0001	0.62–0.80	0.79	<0.0001	0.67–0.92
TTNtv positive	1.76	0.05	1.01–3.08	2.90	0.002	1.48–5.78
LAVi (per 1 ml/m^2^)	1.03	<0.0001	1.02–1.04	1.03	<0.0001	1.02–1.04
LGE present	1.32	0.13	0.92–1.89	—	—	—
FHx DCM	0.59	0.06	0.34–1.00	—	—	—
FHx SCD	0.62	0.09	0.35–1.06	—	—	—

Results of logistic regression model of predictors of early arrhythmias in dilated cardiomyopathy (DCM). Variables with p < 0.10 from the univariable analysis were considered for inclusion in an optimized multivariable model, created using backward stepwise selection until only significant variables remained. Truncating variant in titin (TTNtv) was added to this optimized model.

FHx = family history; LAVi = indexed left atrial volume; LGE = midwall fibrosis late gadolinium enhancement; LVEF = left ventricular ejection fraction; RVEF = right ventricular ejection fraction; SCD = sudden cardiac death.
